# Hydrostatically driven microfluidic sorting of highly motile sperm using multiple parallel microchannels via rheotaxis and thigmotaxis

**DOI:** 10.1007/s10544-026-00858-2

**Published:** 2026-10-29

**Authors:** Toru Hyakutake, Hayato Ikeno, Rinnosuke Takahashi, Manabu Yasui, Masahiro Mita, Shinya Yumoto, Kiyoshi Akiyama, Hayato Tsukahara, Atsushi Shirasawa

**Affiliations:** 1 Faculty of Engineering, Yokohama National University, Kanagawa, Japan; 2 Graduate School of Engineering, Yokohama National University, Kanagawa, Japan; 3 Kanagawa Institute of Industrial Science and Technology, Kanagawa, Japan; 4 Kyodo International Inc, Kanagawa, Japan; 5 Kanagawa Prefectural Livestock Industry Technology Center, Kanagawa, Japan; 6 Zen-noh Embryo Transfer Center, Hokkaido, Japan

**Keywords:** Microfluidic sperm sorting, Rheotaxis, Thigmotaxis, Agent-based modelling, Assisted reproductive technology

## Abstract

Efficient and non-invasive sperm selection is critical for improving outcomes in assisted reproduction and livestock breeding. Herein, we present a hydrostatically driven microfluidic device that exploits intrinsic sperm behaviours, namely rheotaxis and thigmotaxis, to selectively isolate highly motile sperm without external actuation. The device features a multiple parallel-channel architecture designed to increase processing capacity through channel parallelisation while maintaining physiologically relevant conditions. The effects of key geometric and hydrodynamic parameters, including channel numbers, channel spacing, reservoir base aspect ratio, and reservoir volume difference, were systematically investigated through experiments and agent-based numerical simulations. Although increasing the number of channels enhanced sperm recovery, the increase was non-linear owing to recirculation effects induced by thigmotaxis. A cross-shaped channel configuration enabled a maximum recovery efficiency of 2.4%, total recovery of 2.3 × 10⁵ sperm in 30 min, and sperm concentration exceeding 2.3 × 10^6^ cells mL^− 1^. However, simulations revealed that 53% of the sperm reaching the recovery reservoir re-entered the channels, highlighting the importance of suppressing recirculation for further performance improvement. The study findings provide design guidelines for hydrostatically driven, parallel-channel sperm-sorting devices by demonstrating that increasing the channel numbers alone does not necessarily result in a proportional increase in sperm recovery owing to thigmotaxis-mediated re-entry. The proposed platform enables pump-free and centrifugation-free sperm selection with low equipment requirements owing to its passive operation.

**Graphical Abstract:**

**Figure:**
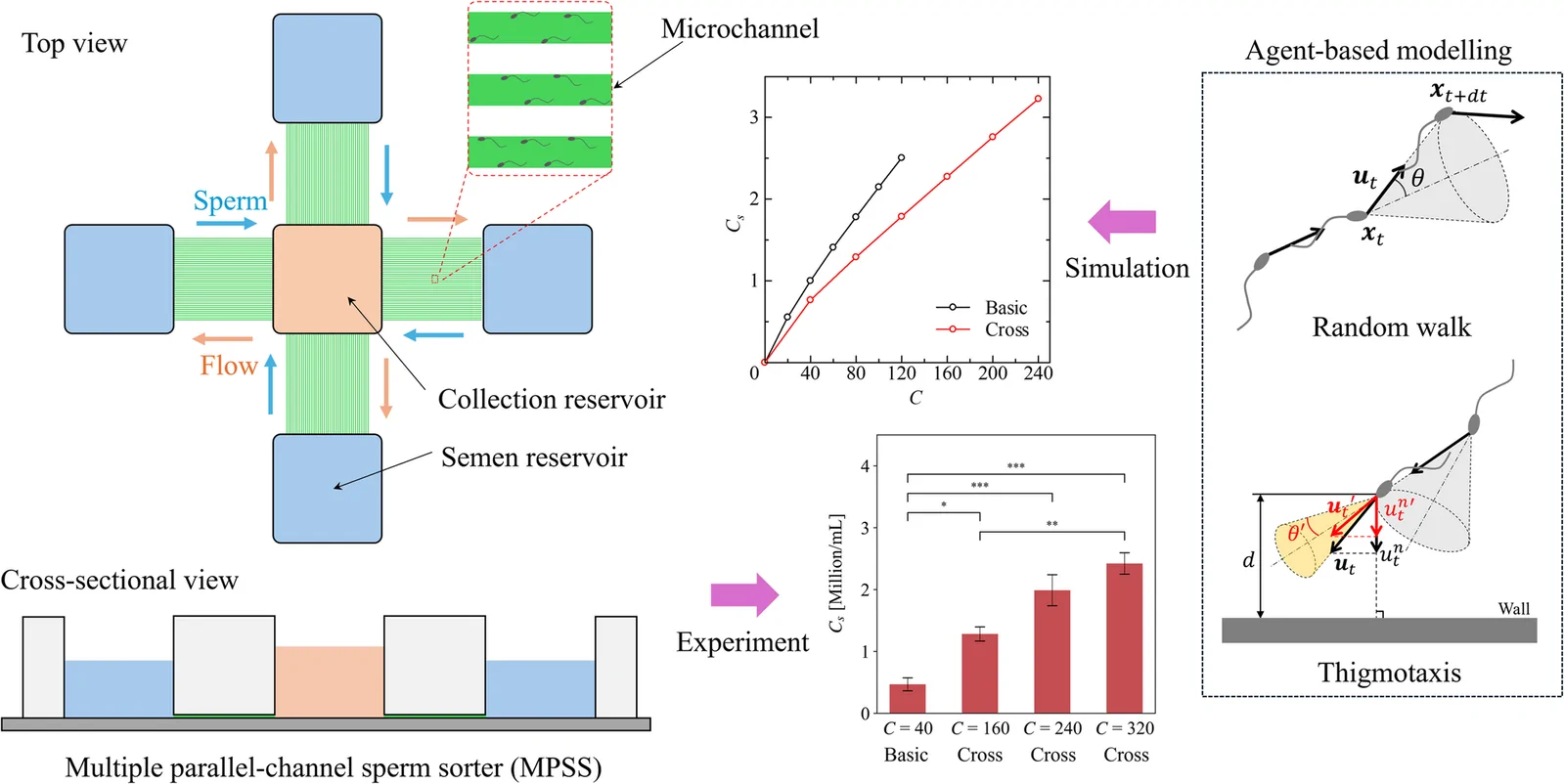

**Supplementary Information:**

The online version contains supplementary material available at https://doi.org/10.1007/s10544-026-00858-2.

## Introduction

Declining birth rates, particularly in developed countries, represent a societal challenge that threatens the stability of social and economic structures. A major contributing factor is infertility. In Japan, more than 20% of couples have undergone infertility testing or treatment, and this proportion has been steadily increasing (National Institute of Population and Social Security Research 2016). Assisted reproductive technologies (ARTs), including in vitro fertilisation (IVF), are widely used to treat infertility, and the proportion of children conceived through IVF has increased markedly over the past two decades (Adamson et al. 2023). Infertility has various causes, with approximately half of all cases attributed to male factors, including impaired sperm motility and reduced sperm count (Choy and Eisenberg 2018). Therefore, the efficient selection of highly motile sperm from semen samples is essential for successful fertilisation. Similar challenges also exist in livestock production systems. In recent years, declining conception rates in breeding cattle have become a significant concern (Lucy 2001; Irikura et al. 2018; Dochi et al. 2010). Notably, conception rates following embryo transfer using in vitro-produced embryos remain lower than those achieved through artificial insemination (Ealy 2019). The resulting increase in the number of open days leads to substantial economic losses for farmers and poses a serious threat to farm sustainability. One factor contributing to reduced fertility in both human ARTs and livestock breeding applications is the use of conventional sperm preparation techniques, such as density gradient centrifugation (DGC). These methods involve centrifugation processes that can induce physical stress in spermatozoa, leading to DNA damage (Muratori et al. 2019), which has been associated with impaired embryo development and reduced reproductive outcomes (Simon et al. 2013; Lourenço et al. 2023). Accordingly, technologies capable of selectively isolating highly motile sperm under physiologically relevant conditions, while simultaneously increasing the yield of sorted sperm, are urgently needed.

Microfluidic technologies have attracted considerable attention owing to their ability to precisely manipulate fluids and suspended particles at the microscale. Recent advances in microfluidic engineering have enabled a wide range of biomedical applications through the development of innovative device architectures and flow-control strategies for particle and cell manipulation, separation, and sorting (Mansor et al. 2025; Han et al. 2025; Tian et al. 2024; Cui et al. 2025; Chen et al. 2025). Among these applications, microfluidic technologies have been extensively investigated as promising alternatives to conventional sperm selection methods such as swim-up and DGC. Early studies demonstrated that passively driven microfluidic systems can separate motile sperm based on their ability to actively swim through microchannels (Cho et al. 2003; Schuster et al. 2003). Nosrati et al. developed microfluidic devices capable of rapidly isolating sperm with high DNA integrity by exploiting differences in motility (Nosrati et al. 2014). Asghar et al. proposed a membrane-based microfluidic platform, inspired by the conventional swim-up method, enabling the selective recovery of sperm with reduced DNA damage and lower levels of reactive oxygen species (Asghar et al. 2014). Microfluidic devices incorporating pillar-array structures have also been introduced (Chinnasamy et al. 2018), creating physical environments that selectively permit the passage of sperm with high motility and progressive movement. Conversely, sperm exhibit a characteristic response to fluid flow known as rheotaxis (Miki and Clapham 2013; Kantsler 2014; El-Sherry et al. 2014; Zhang 2016; Rappa et al. 2018). This behaviour has been increasingly exploited in microfluidic systems, where controlled flow fields enable the selective isolation of highly motile sperm (Seo et al. 2007; Nagata et al. 2018; Zaferani et al. 2018; Sarbandi et al. 2021; Aguirregomezcorta 2021; Atael et al. 2021; Sharma et al. 2022). Seo et al. developed a hydrostatically driven microfluidic device that utilises rheotaxis to achieve efficient sperm selection under flow conditions (Seo et al. 2007). Nagata et al. demonstrated that the motility patterns of sperm selected using microfluidic devices correlate with fertilisation potential and have been successfully applied in artificial insemination (Nagata et al. 2018). Sarbandi et al. introduced a biomimetic microfluidic device with micro-pocket structures that generate favourable hydrodynamic conditions for guiding highly motile sperm (Sarbandi et al. 2021). Romero-Aguirregomezcorta showed that rheotaxis-based selection improves sperm quality and enhances early embryo development (Aguirregomezcorta 2021). Additionally, sperm exhibit a tendency to swim along surfaces, a behaviour known as thigmotaxis (Woolley 2003; Elgeti 2010; Nosrati et al. 2016). More recently, several studies have reported microfluidic sperm selection systems that combine rheotaxis and thigmotaxis, leading to improved sperm selection performance (Ahmadkhani et al. 2023; Zeaei et al. 2023; Bouloorchi 2024; Asl et al. 2024; Puri 2026). The use of sperm selected based on these intrinsic behaviours increases blastocyst formation rates (Yaghoobi et al. 2024). Several studies have highlighted the importance of microchannel geometry in mimicking natural selection processes within the female reproductive tract, demonstrating its significant impact on sperm selection efficiency (Zaferani et al. 2019; Heydari et al. 2023; Vasilescu et al. 2023).

Despite these advances, several limitations remain in existing microfluidic sperm selection devices. First, many systems yield relatively low sperm concentrations, limiting their practical applicability and scalability. Second, most rheotaxis-based devices rely on external flow control using syringe pumps, increasing system complexity and restricting their use in clinical and field settings. Consequently, only a limited number of these devices have been successfully actualised into practical or commercial applications.

To address these challenges, we propose a microfluidic device that enables the non-invasive selection and sorting of highly motile sperm by exploiting intrinsic sperm behaviours, namely rheotaxis and thigmotaxis, within an appropriate flow field generated by hydrostatic pressure. Hydrostatic actuation enables pump-free operation without an external power supply. The device features a multiple parallel-channel architecture, termed the multiple parallel-channel sperm sorter (MPSS), in which microchannels with dimensions comparable to sperm lengths are arranged in parallel. This architecture is designed to increase sperm processing capacity through channel parallelisation while maintaining pump-free operation. The principal advances of the present study are the introduction of a multiple parallel-channel architecture and systematic investigation of how channel parallelisation and device geometry interact with rheotaxis and thigmotaxis to govern sperm recovery. Specifically, we evaluated the effects of key geometric parameters, including channel numbers, channel spacing, and reservoir geometry, through a combination of experiments and numerical analyses. The analyses aimed to provide mechanistic insights into sperm transport and establish design guidelines for hydrostatically driven, parallel-channel sperm sorting.

## Materials and methods

### Device fabrication and geometrical design

Microfluidic channels were fabricated in polydimethylsiloxane (PDMS) using standard soft-lithography techniques (Duffy et al. 1998). The master mould was prepared on a silicon wafer using photolithography with a 10-µm-thick SU-8 photoresist (SU-8 3010, Nippon Kayaku Co., Tokyo, Japan). The patterned wafer was subsequently subjected to dry etching to produce a silicon mould, followed by removal of the residual photoresist. The PDMS prepolymer and curing agent (Sylgard 184 Silicone Elastomer Kit, Dow Corning Toray Co., Tokyo, Japan) were mixed at a ratio of 10:1, degassed, and poured onto the SU-8 master mould. After curing, the PDMS replica was carefully peeled from the mould. The inlet and outlet ports were then created using a custom rectangular punch to ensure a uniform channel length across multiple parallel channels. Finally, the PDMS substrate and a glass slide were treated with oxygen plasma and irreversibly bonded to form the microfluidic device.

**Fig. 1:**
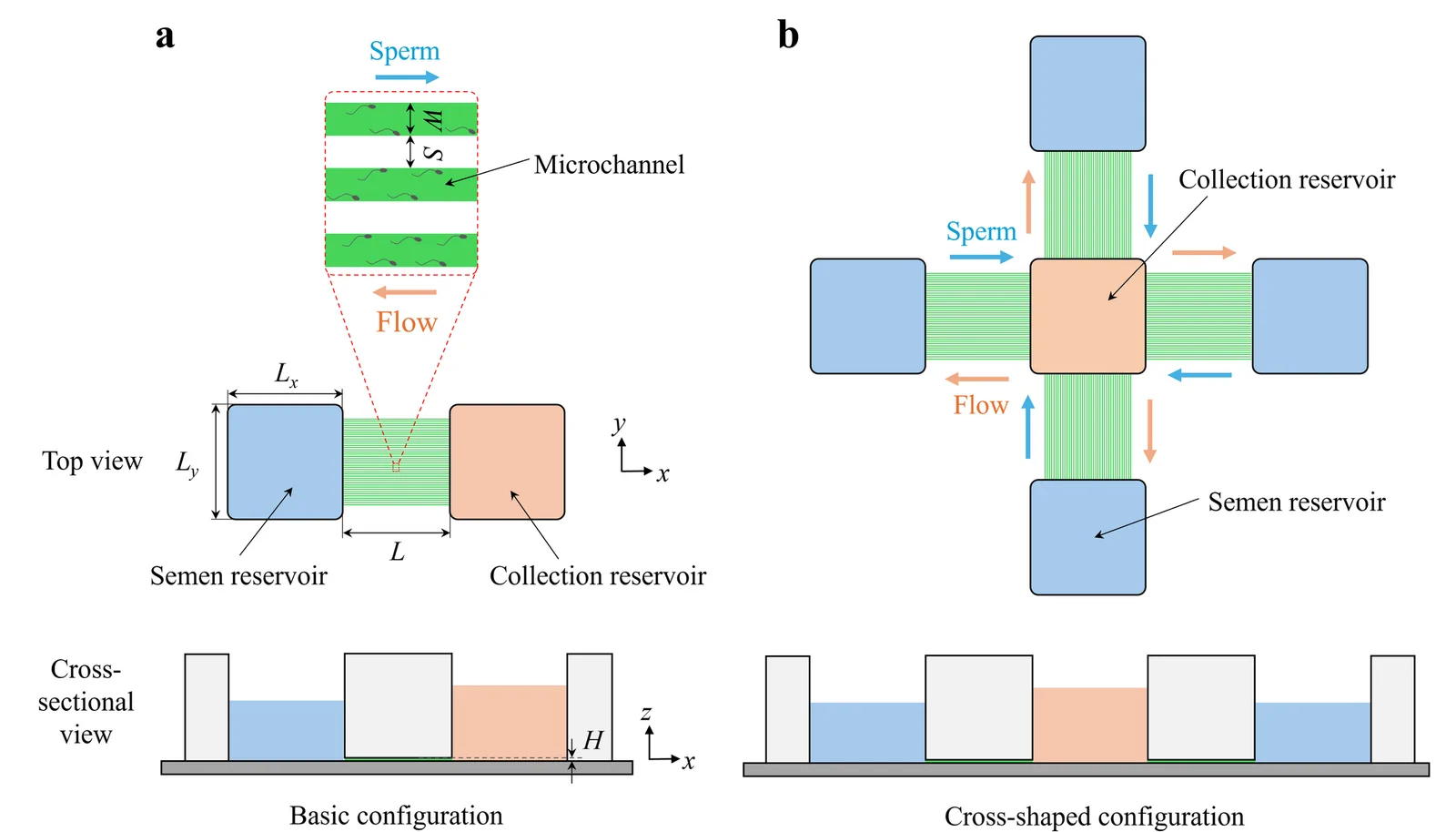
Schematics of the microfluidic devices used in this study. (**a**) Basic configuration comprising a single semen reservoir and single collection reservoir connected by multiple parallel microchannels. (**b**) Cross-shaped configuration with four semen reservoirs connected to a central collection reservoir via multiple parallel microchannels. The device is fabricated using soft lithography with polydimethylsiloxane (PDMS) and bonded to a glass substrate. The channel length (*L*), width (*W*), and height (*H*) are fixed

As shown in Fig. 1, two types of microfluidic devices were designed. The first, referred to as the basic configuration, comprised a single semen reservoir connected to a single collection reservoir through multiple parallel microchannels. The second, referred to as the cross-shaped configuration, comprised four semen reservoirs connected to a single collection reservoir via multiple parallel microchannels. In both configurations, the channel length *L*, width *W*, and height *H* were fixed at 5 mm, 50 μm, and 10 μm, respectively. The effects of geometric parameters, including the number of channels *C*, channel spacing *S*, and reservoir geometry, on the sperm concentration in the collection reservoir were systematically investigated. For the basic configuration, three channel-number conditions were tested (*C* = 40, 80, and 120), whereas for the cross-shaped configuration, three conditions were examined (*C* = 160, 240, and 320), with each side of the collection reservoir connected to a semen reservoir through 40, 60, or 80 channels, respectively. The channel spacing in the basic configuration was set to *S* = 17, 50, and 100 μm. For the reservoir geometry, the shorter side of the reservoir base was denoted by *L*_*x*_, whereas the longer side connected to the microchannels was denoted by *L*_*y*_. The aspect ratio was defined as *Γ = L*_*y*_/*L*_*x*_. In the basic configuration, the reservoir base area ($\:{A}_{\mathrm{r}\mathrm{e}\mathrm{s}}$ = *L*_*x*_ *L*_*y*_) was kept constant, while three aspect ratios were examined (*Γ* = 1, 2.34, and 5.43). The effect of the initial volume difference (Δ*V)* between reservoirs on sperm concentration in the collection reservoir was also investigated.

### Sperm-sorting experiments and analysis

Frozen semen (0.5 mL) from a Japanese Black bull (*Shigeharukuni*; LIAJ, Maebashi, Japan) was thawed. The collection reservoir and microchannels were first filled with BO medium (IFP963B, Research Institute for the Functional Peptides, Yamagata, Japan) supplemented with 0.6 wt% bovine serum albumin. The prepared sperm suspension was then introduced into the semen reservoir. The volume difference between the collection and semen reservoirs generated an appropriate flow from the collection reservoir to the semen reservoir via hydrostatic pressure. Under these flow conditions, motile sperm migrated upstream towards the collection reservoir via rheotaxis. The device was subsequently incubated for 30 min, after which fluid from the collection reservoir was collected. All experiments were conducted at 38.5 °C. The collected samples were analysed using a sperm motility analysis system (SMAS). The evaluated parameters included recovered sperm concentration (*C*_*s*_), curvilinear velocity (VCL), straightness (STR), amplitude of lateral head displacement (ALH), and beat cross frequency (BCF).

### Statistical analysis

Each experiment was performed with *N* = 4 independent replicates, with each replicate representing an independent semen preparation from a separate straw obtained from the same bull. Statistical analyses were performed using Student’s *t*-test for two-group comparisons, including comparisons between the semen and collection reservoirs and comparisons of various initial volume differences. For all other comparisons, one-way analysis of variance (ANOVA) followed by Tukey’s honestly significant difference post hoc test was used. Before applying parametric statistical analyses, normality and homogeneity of variance were assessed using the Shapiro–Wilk and Levene’s tests, respectively. Effect sizes were calculated as Cohen’s *d* for Student’s *t*-tests and η² for one-way ANOVA. Differences were considered statistically significant at *p* < 0.05. Detailed statistical results are provided in Table S2 and S3.

### Theoretical analysis of hydrostatically driven flow

Under hydrostatic driving conditions, the flow rate in the microchannels varied with time owing to the decay of the volume difference between the reservoirs. Therefore, estimation of the temporal evolution of the flow in advance was important. Because the microchannels had a rectangular cross-section, the channel geometry was modelled as a rectangular duct with width 2*a* and height 2*b*. The cross-sectional mean velocity $\:{u}_{m}\left(t\right)$ in a rectangular microchannel can be approximated from the axial velocity distribution as follows (White 1991):

1 $$\begin{aligned}&\;\;u_{m}(t)=\frac{\rho{ga}^{2}}{3{\mu}L}F(a, b)\triangle{h}(t)\\&F(a, b)=1-\frac{192a}{b\pi^{5}}\sum_{n=1, 3, 5,...}^{\infty}\frac{1}{n^{5}}\:tanh\:\frac{n{\pi}b}{2a}\end{aligned}$$

where Δ*h*, $\:\rho\:$, and *g* denote the hydrostatic head difference, fluid density, and gravitational acceleration, respectively. The relationship between the volume difference Δ*V* and the corresponding hydrostatic head difference Δ*h* is given by:

2 $$\:{\Delta\:}h\left(t\right)=\frac{{\Delta\:}V\left(t\right)}{{A}_{\mathrm{r}\mathrm{e}\mathrm{s}}\:}$$

Fluid flows through *C* parallel microchannels; therefore, the temporal evolution of the volume difference can be expressed as:

3 $$\:\frac{d{\Delta\:}V\left(t\right)}{dt}=\:-2CQ\left(t\right)=\:-C\frac{8\rho\:g{a}^{3}b}{3\mu\:L{A}_{\mathrm{r}\mathrm{e}\mathrm{s}}}F\left(a,b\right){\Delta\:}V\left(t\right)$$

Solving this equation yields the following expression for the mean velocity:

4 $$\:{u}_{m}\left(t\right)=\left(\frac{\rho\:g{a}^{2}}{3\mu\:L}\frac{{\Delta\:}V}{{A}_{\mathrm{r}\mathrm{e}\mathrm{s}}}F\left(a,b\right)\right)\mathrm{e}\mathrm{x}\mathrm{p}\left[-t\frac{8C\rho\:g{a}^{3}b\:F\left(a,b\right)}{3\mu\:L{A}_{\mathrm{r}\mathrm{e}\mathrm{s}}}\right]$$

where Δ*V* denotes the volume difference at *t* = 0. Equation (4) was used to predict the time-dependent variation in the mean flow velocity in the microchannels as a function of channel number *C*.

### Agent-based modelling of sperm swimming

**Fig. 2:**
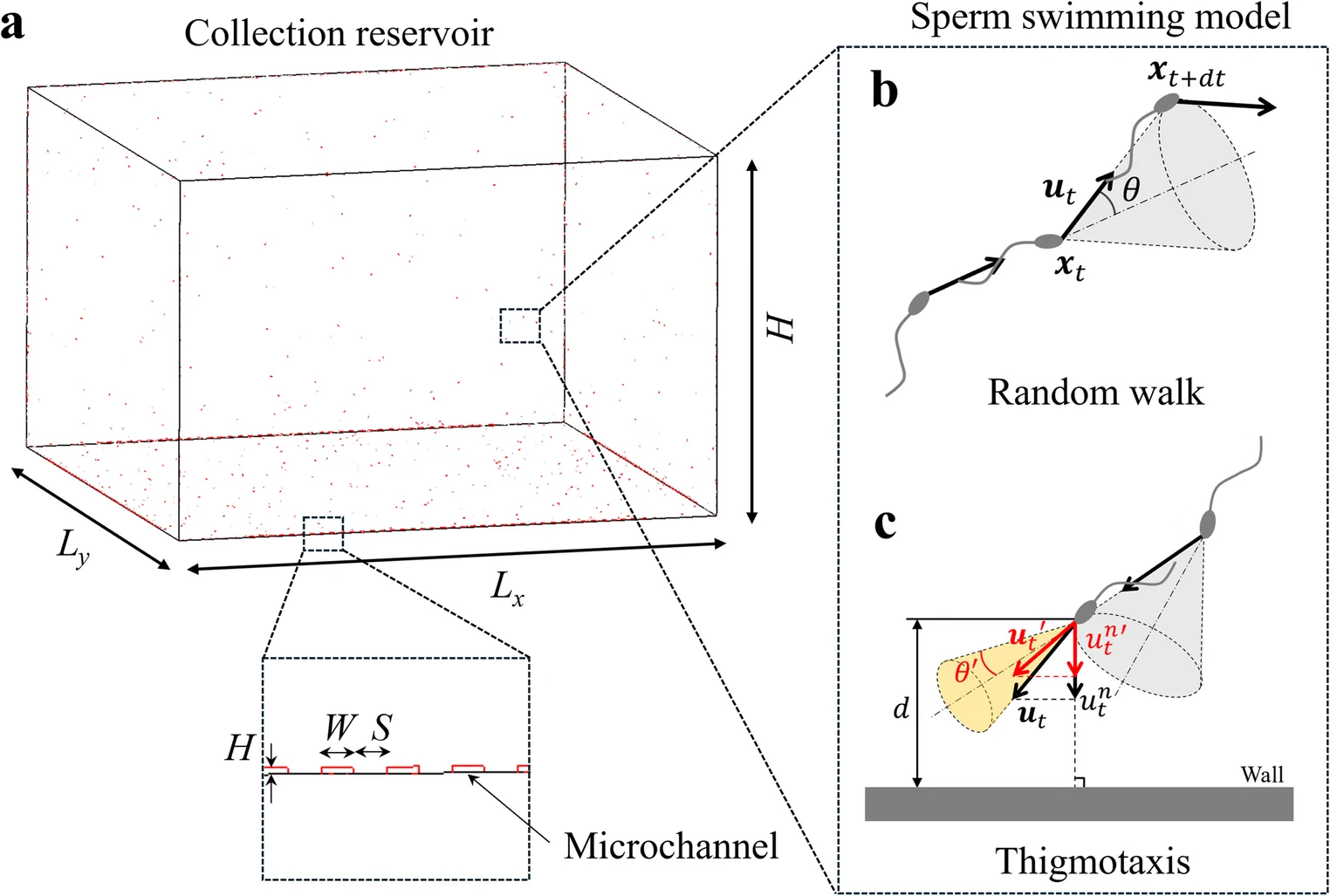
Overview of the agent-based model (ABM) used to simulate sperm swimming behaviour. (**a**) Computational domain corresponding to the collection reservoir, with channel inlets located at the boundaries. (**b**) Schematic of sperm motion modelled as a stochastic random walk with 3D variation in swimming direction. (**c**) Representation of thigmotaxis, in which sperm preferentially swim along surfaces, leading to accumulation near walls and corners

Experimental studies using microfluidic devices require systematic evaluation under a wide range of conditions, which can be time-consuming and labour-intensive. Therefore, numerical simulations provide an effective approach for optimising experimental conditions and improving research efficiency. An agent-based model (ABM) was employed to predict sperm swimming behaviour. In this approach, individual spermatozoa are treated as discrete agents, and their collective dynamics are described probabilistically, enabling the analysis of sperm motion at spatiotemporal scales comparable to those of microfluidic devices and the female reproductive tract (Kromer et al. 2018; Zaferani et al. 2019; Diemer et al. 2021; Hyakutake et al. 2024). As illustrated in Fig. 2, the collection reservoir was selected as the computational domain. ABM simulations were conducted to evaluate the effects of reservoir geometry and the number and arrangement of microchannels on sperm swimming behaviour within the collection reservoir.

In this study, sperm were represented as point-like agents. Typically, sperm swim along zigzag trajectories driven by flagellar beating. This behaviour was approximated using a stochastic random-walk model (Fig. 2). In the model, the propulsion direction of each sperm was defined by a deflection angle $\:\theta\:$, which evolves in three dimensions at each time step. The deflection angle $\:\theta\:$ was assumed to follow a normal distribution with a mean of 0 and standard deviation $\:{\theta\:}_{\mathrm{s}\mathrm{d}}$, which was determined from the sperm trajectories measured for the recovered sperm in the present experiments. Its value was updated at each time step using random sampling. Similarly, the swimming speed was sampled from a normal distribution characterised by the mean swimming velocity $\:{V}_{s}^{\mathrm{a}\mathrm{v}\mathrm{g}}$ and standard deviation $\:{V}_{s}^{\mathrm{s}\mathrm{d}}$, which were determined from the swimming velocities of the recovered sperm measured in the present experiments. To account for thigmotaxis, that is, the tendency of sperm to swim along surfaces, a phenomenological model was introduced in which both the wall-normal velocity component $\:{u}_{t}^{n}$ and the deflection angle $\:\theta\:$ reduced as the distance from the wall decreased (Hyakutake et al. 2024). This behaviour was modelled as:

5 $$u^{n\prime}_{t}\:u^{n}_{t}\:(1-e^{-k_{t}d})$$

6 $$\theta^{\prime}=\theta\:(1-e^{k_{t}d})$$

where *d* is the distance from the wall, and $\:{k}_{t}$ is a parameter that quantifies the strength of thigmotaxis. The value of $\:{k}_{t}$ was adopted from a study by Hyakutake et al. (2024), in which $\:{k}_{t}$ was calibrated to reproduce the experimental observations of sperm thigmotaxis reported by Nosrati et al. (2016). Based on Eq. (5), the wall-normal velocity component was updated to $\:{{u}_{t}^{n}}^{{\prime\:}}$, from which the new direction vector $\:{(\mathbf{u}}_{t}^{{\prime\:}}$) was calculated. The sperm position at the next time step ($\:{\mathbf{x}}_{t+{\Delta\:}t}$) was then updated explicitly. The tangential velocity component ($\:{u}_{t}^{t})$ was assumed to remain unchanged. This formulation enabled the degree of thigmotaxis to be controlled using a single parameter ($\:{k}_{t}$) while reproducing the characteristic accumulation of sperm near the surface.

As shown in Fig. 2, the collection reservoir was modelled as a rectangular cuboid with dimensions $\:{L}_{x}\times\:{L}_{y}\times\:H$, where the height * H* was adjusted such that the total volume matched the experimental conditions. The shape of the liquid meniscus was not considered in this study. At the bottom surface, the interfaces connecting the microchannels with width (*W*) and height (*H*) were defined. Sperm were introduced into the collection reservoir through the channel interfaces at a rate of one sperm per time step from each side of the collection reservoir. Because the simulation time step was 0.05 s and total simulation time was 30 min (1800 s), a total of 36,000 sperm were introduced from each side during one simulation. Accordingly, the total number of introduced sperm were 36,000 and 144,000 for the basic and cross-shaped configurations, respectively. The channel spacing was set to *S*. In the basic configuration, the microchannels were located on one side of the reservoir, whereas in the cross-shaped configuration, they were arranged on four sides. In both cases, the channels were positioned symmetrically with respect to the centreline of each side. Bulk flow within the collection reservoir was not considered. Sperm motion was governed by the random-walk model described above, with thigmotaxis activated near the walls. Sperm exiting the reservoir through the channel interfaces were removed from the computational domain. In the simulations, the number of sperm present in the collection reservoir was recorded as a function of time. All simulations were performed using five independent runs with different random seeds, and the reported results represent the averages of these simulations. The effects of channel numbers, channel spacing, and reservoir base aspect ratio on sperm accumulation were evaluated. Comparisons were made between the basic and cross-shaped configurations and between cases with and without thigmotaxis. For quantitative comparisons, the sperm concentration in the collection reservoir was normalised by the value obtained under the reference conditions (basic configuration with *C* = 40).

## Results

Under hydrostatic driving conditions, the flow rate in the microchannels gradually decreases with time as the volume difference between the reservoirs decays. Therefore, we first investigated the effect of channel numbers on the temporal evolution of the mean flow velocity using theoretical analysis.

**Fig. 3:**
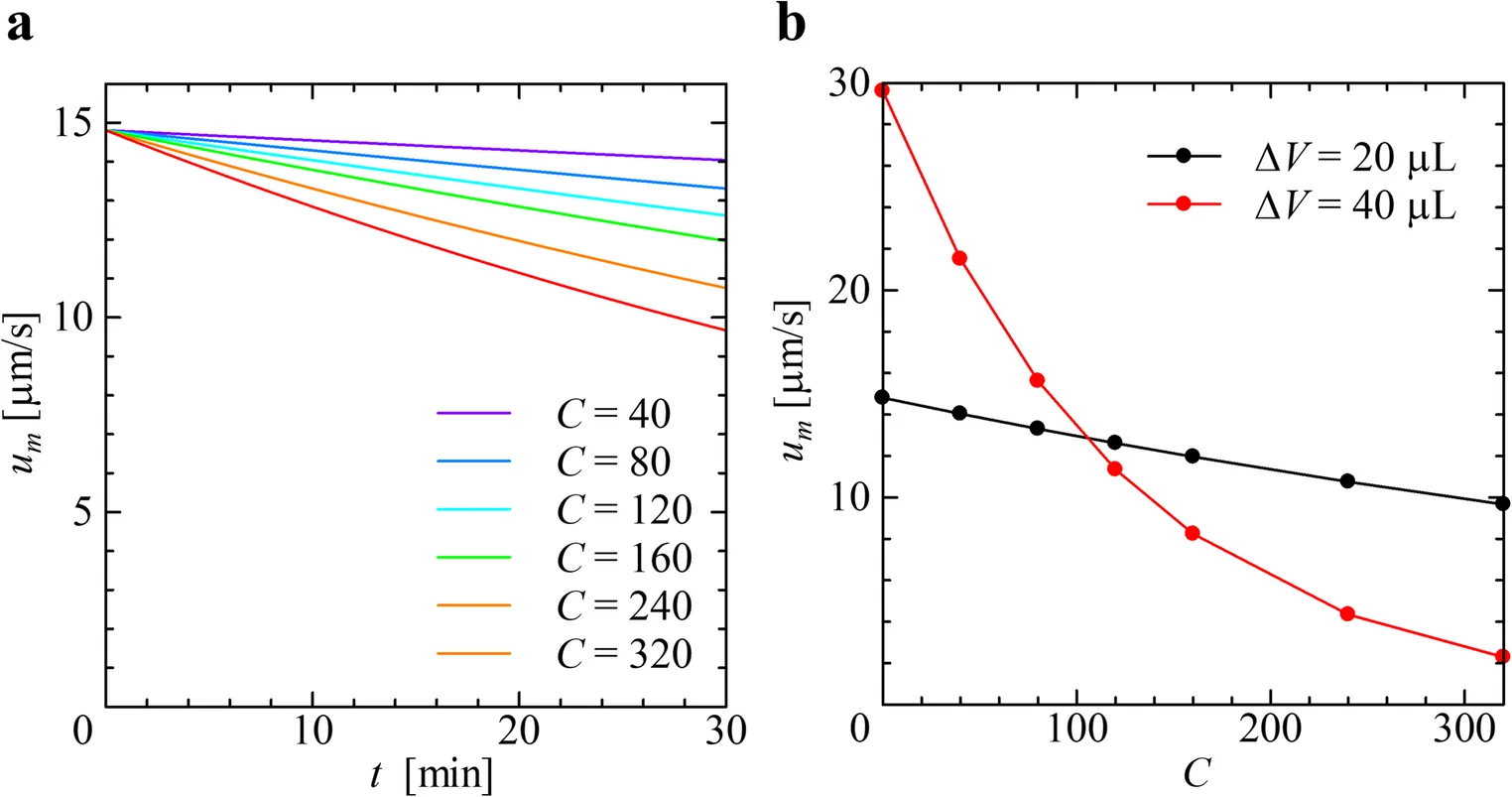
Theoretical prediction of the time-dependent flow velocity in the microchannels. (**a**) Temporal evolution of the mean velocity $\:{u}_{m}\left(t\right)$ for various channel numbers (*C*) with an initial volume difference of Δ*V* = 20 µL. (**b**) Comparison of the mean velocity after 30 min for various channel numbers and initial volume differences. Increasing * C*accelerates the decay of flow velocity

The time-dependent mean velocity $\:{u}_{m}\left(t\right)$ in the microchannels, calculated from Eq. (4) with an initial volume difference of Δ*V* = 20 µL, is shown in Fig. 3(a). The calculation conditions were as follows: fluid temperature *T* = 38.5 °C; reservoir base area *A*_res_ = 28.6 mm^2^; channel width *W* = 50 μm; channel height *H* = 10 μm; and channel length *L* = 5 mm. As time progressed, the mean velocity gradually decreased owing to the reduction in volume difference. For *C* = 40, the velocity decreased by 5.2% after 30 min, whereas for *C* = 320, a larger reduction of 34.7% was observed. These results indicate that increasing the number of channels leads to a greater reduction in flow velocity over time. Figure 3(b) compares the mean velocities after 30 min under two different initial volume differences. The horizontal axis represents the number of channels. A higher initial volume difference resulted in a stronger dependence on channel numbers. For example, at Δ*V* = 40 µL, the velocity reduction was 27.4% for *C* = 40 and approached 90% for *C* = 320.

**Fig. 4:**
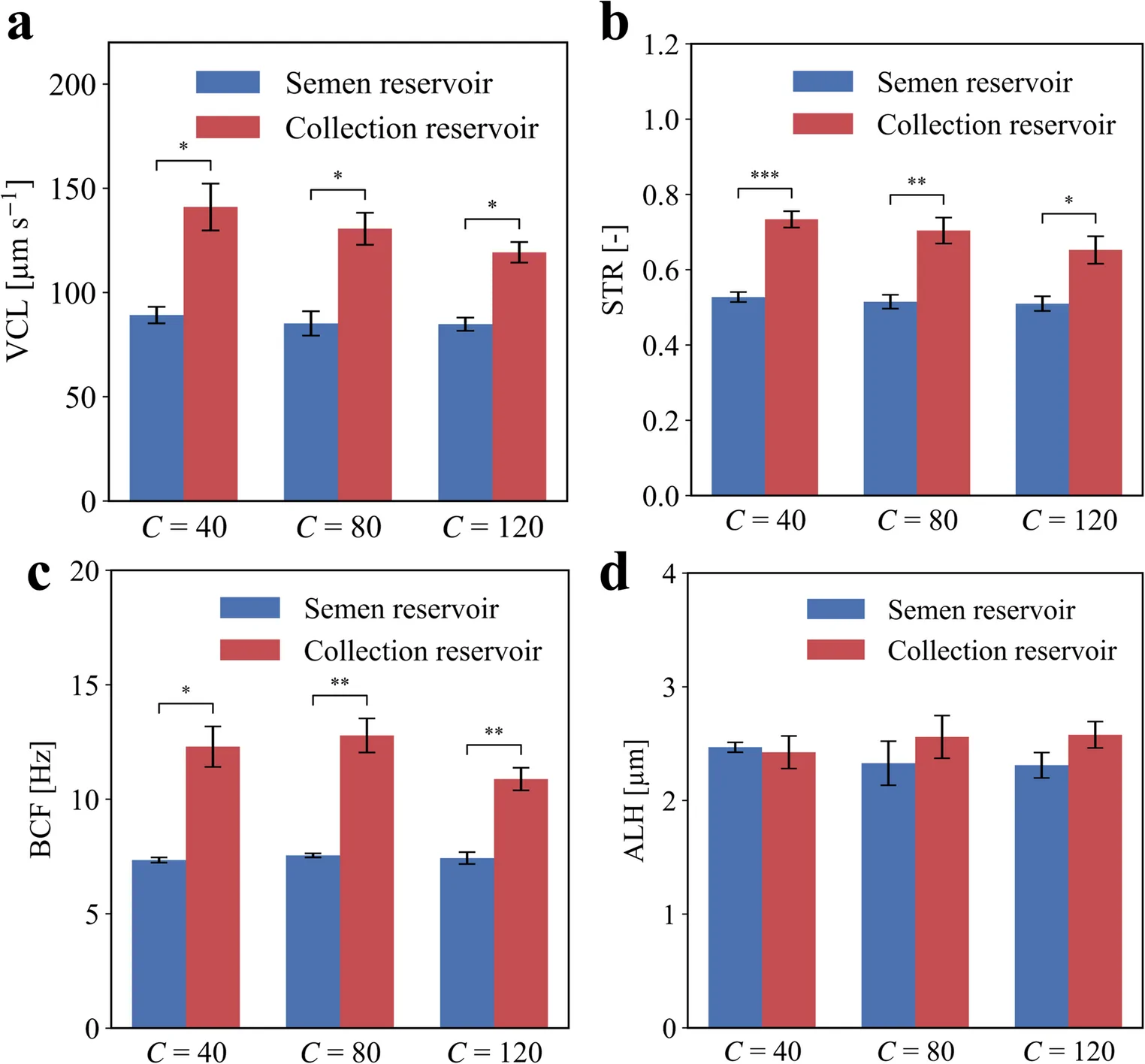
Comparison of sperm motility parameters between the semen and collection reservoirs after 30 min, as measured using SMAS. The evaluated device was the basic configuration with a reservoir base aspect ratio of *Γ* = 5.43. (**a**) VCL, (**b**) STR, (**c**) BCF, and (**d**) ALH. Results are shown for various channel numbers (*C* = 40, 80, and 120) in the basic configuration. Error bars represent standard errors (*N* = 4)

Next, the ability of the device to selectively recover highly motile sperm was evaluated. Figure 4 compares the sperm motility parameters between the semen and collection reservoirs after 30 min. Figure 4(a)–(d) show the VCL, STR, BCF, and ALH, respectively, as measured using the SMAS. The evaluated device was the basic configuration with a reservoir base aspect ratio of *Γ* = 5.43. Owing to its elongated geometry (*L*_*y*_ > *L*_*x*_), multiple parallel channels could be connected between the semen and collection reservoirs. The channel spacing was fixed at *S* = 50 μm, and the initial volume difference was Δ*V* = 20 µL. Comparisons were performed for three channel-number conditions (*C* = 40, 80, and 120), with *N* = 4 independent measurements for each condition. Significant differences between the semen and collection reservoirs were observed for VCL, STR, and BCF under all conditions. By contrast, ALH showed no significant difference between the two reservoirs. With increasing channel numbers, VCL and STR were marginally reduced in the collection reservoir.

**Fig. 5:**
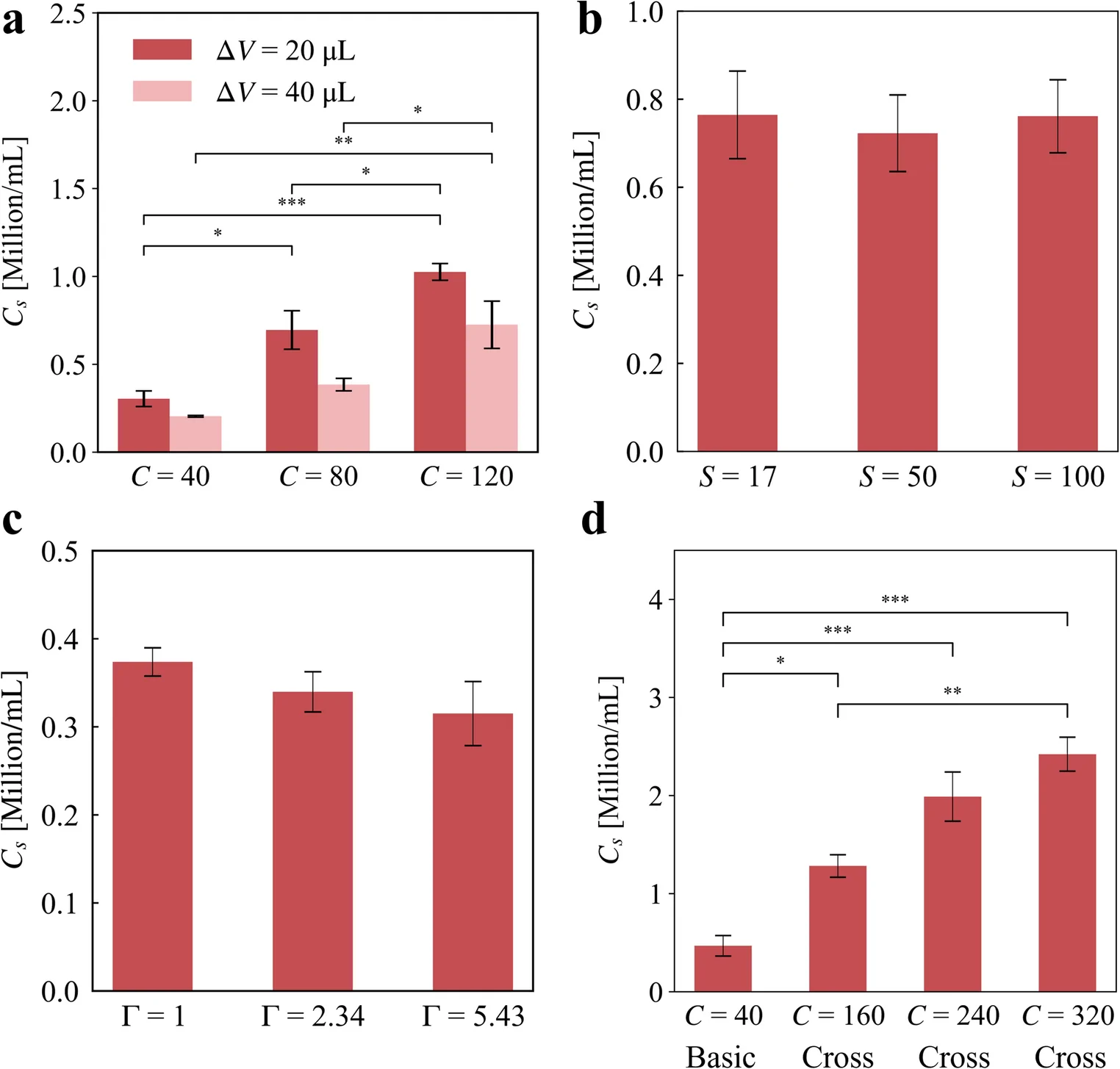
Effects of geometric parameters on sperm concentration. (**a**) Recovered sperm concentration (*C*_*s*_) as a function of channel number *C* for various initial volume differences. (**b**) Effect of channel spacing (*S*) on sperm concentration. (**c**) Effect of reservoir base aspect ratio (*Γ)* on sperm concentration. (**d**) Comparison of sperm concentration between the basic and cross-shaped configurations. Error bars represent standard errors (*N* = 4)

The effects of geometric parameters on recovered sperm concentration were then evaluated. Figure 5(a) shows a comparison of the sperm concentration *C*_*s*_ under three channel-number conditions in the basic configuration, with *Γ* = 5.43 and *S* = 50 μm. Additionally, two initial volume differences were examined. As shown in Fig. 5(a), the recovered sperm concentration increased with increasing channel numbers. A smaller initial volume difference resulted in a higher recovered sperm concentration. The effects of channel spacing *S* and reservoir base aspect ratio *Γ* are presented in Fig. 5(b) and (c), respectively. To evaluate the effect of channel spacing, the channel number and aspect ratio were fixed at *C* = 80 and *Γ* = 5.43, respectively. Conversely, to evaluate the effect of aspect ratio, the channel number and channel spacing were fixed at *C* = 40 and *S* = 50 μm, respectively. As shown in Fig. 5(b), no significant difference in the sperm concentration was observed among the different channel spacings. However, as shown in Fig. 5(c), sperm concentration exhibited a marginally decreasing trend with increasing aspect ratio, although no statistically significant difference was detected.

The performance of the cross-shaped configuration was further evaluated by comparison with that of the basic configuration. The reference condition was the basic configuration with *C* = 40 and *Γ* = 1. In the cross-shaped configuration using four semen reservoirs, as shown in Fig. 1, each reservoir was connected through *C* = 40, 60, or 80 channels. Accordingly, the total number of channels in the devices were *C* = 160, 240, and 320, respectively. Figure 5(d) shows a comparison of recovered sperm concentrations between the basic and cross-shaped configurations. With increasing total channel number *C*, recovered sperm concentration also increased. However, this increase was not proportional to *C*. Compared with the basic configuration (*C* = 40), the cross-shaped configurations (*C* = 160, 240, and 320), corresponding to 4-, 6-, and 8-fold increases in channel numbers, resulted in approximately 2.3-, 3.2-, and 4.1-fold increases in sperm concentration, respectively.

**Fig. 6:**
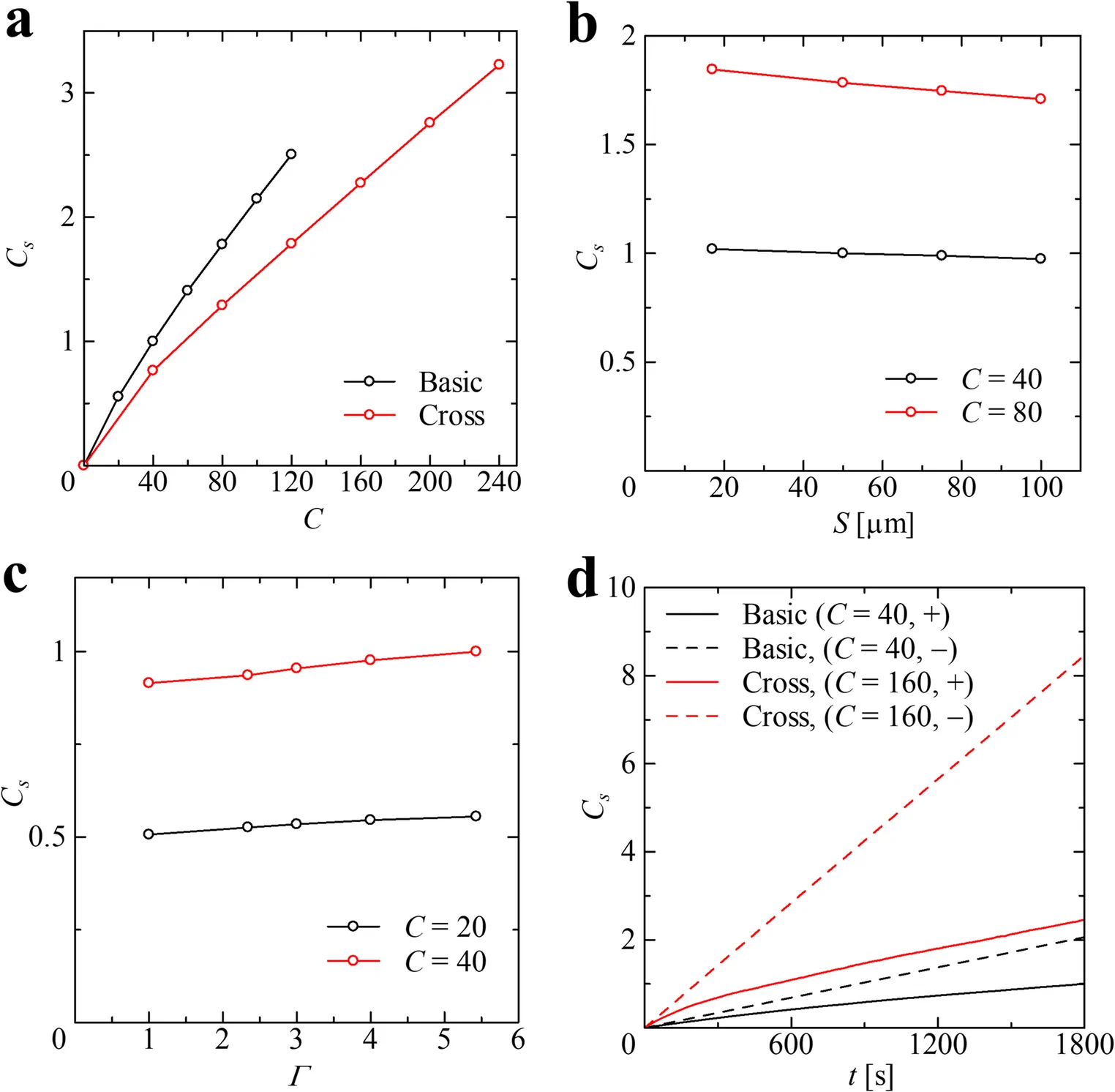
Numerical simulation results of sperm concentration based on the agent-based model (ABM). (**a**) Normalised sperm concentration as a function of channel number *C* for the basic and cross-shaped configurations. (**b**) Effect of channel spacing (*S*) on sperm concentration. (**c**) Effect of reservoir base aspect ratio (*Γ*) on sperm concentration. (**d**) Temporal evolution of the sperm concentration in the collection reservoir with and without thigmotaxis (+ and − denote the presence and absence of thigmotaxis, respectively). Inclusion of thigmotaxis reduces sperm accumulation owing to recirculation effects

Figure 6 shows the numerical simulation results. Here, $V_{S}^{avg},\:\theta_{\mathrm{sd}}$, and $k_{t}$ were set to 120 μm s^-1^, 25°, and 0.15, respectively, to match the experimental conditions. Fig. 6(a) shows the normalised sperm concentration in the collection reservoir as a function of channel number *C*. The channel dimensions were fixed at *W* = 50, *S* = 50, and *H* = 10 μm, with a reservoir base aspect ratio of *Γ* = 1. The _s_perm concentration *C*_*s*_ was normalised by the value obtained for the basic configuration at *C* = 40. Sperm concentration increased with increasing channel numbers; however, this increase was not proportional. For the same number of channels, the cross-shaped configuration exhibited a lower sperm concentration than the basic configuration. Fig. 6(b) shows the effect of channel spacing *S* on the basic configuration. The channel width and height were fixed at *W* = 50 and *H* = 10 μm, respectively, with *Γ* = 1. A marginal decrease in sperm concentration was observed with increasing channel spacing. Fig. 6(c) shows the effect of the reservoir base aspect ratio Γ for the basic configuration, with *W* = 50, *S* = 50, and *H* = 10 μm. Sperm concentration exhibited a minimal increasing trend with increasing aspect ratio. Fig. 6(d) shows the temporal evolution of sperm concentration in the collection reservoir for the basic (*C* = 40) and cross-shaped configurations (*C* = 160). In all cases, the sperm concentration increased over time. When thigmotaxis was not considered, the sperm concentration increased approximately linearly with time. By contrast, the inclusion of thigmotaxis resulted in significantly lower sperm concentrations than those observed in cases without thigmotaxis. Specifically, the final sperm concentration decreased by approximately 50% in the basic and 70% in the cross-shaped configuration.

## Discussion

A microfluidic device was developed to enable the efficient selection and recovery of highly motile sperm by exploiting intrinsic sperm behaviours, namely rheotaxis and thigmotaxis. Although hydrostatic actuation has been employed in previous microfluidic sperm-sorting devices, the principal advances of the present study lie in the multiple parallel-channel architecture and systematic evaluation of how channel and reservoir geometry influence sperm recovery through the interplay between rheotaxis and thigmotaxis. By designing the channel width to be comparable to the sperm body length, the device facilitated boundary-following behaviour, thereby enhancing the contribution of thigmotaxis. An appropriate flow field driven by a hydrostatic pressure difference was sustained for a sufficient duration, enabling the stable induction of rheotaxis and selective upstream migration of highly motile sperm. The results demonstrated that increasing the number of microchannels enhanced the recovered sperm concentration. Extending the device architecture from the basic configuration to a cross-shaped configuration increased the total number of channels and improved recovered sperm concentration to a maximum of $\:2.4\times\:{10}^{6}$ cells mL^− 1^. However, the increase in sperm recovery was not proportional to the increase in channel numbers. The experimental observations and numerical analyses demonstrated that sperm reaching the collection reservoir can subsequently re-enter the microchannels as a consequence of thigmotactic boundary-following behaviour. This re-entry reduces net sperm accumulation in the collection reservoir and, therefore, limits the expected benefit of increasing the number of parallel channels. This finding identifies an important design constraint for parallelised microfluidic sperm-sorting systems and indicates that increasing the channel numbers alone is insufficient to achieve a proportional increase in sperm recovery. Accordingly, the present study provides mechanistic insight into the relationship between device geometry and sperm transport and establishes design considerations for improving sperm recovery by balancing channel parallelisation with sperm re-entry suppression.

The performance of the proposed microfluidic device is strongly governed by the geometric and hydrodynamic parameters, including channel numbers, reservoir base aspect ratio, channel cross-section, and volume difference between the reservoirs. Increasing the number of channels generally enhances recovered sperm concentration. This outcome can be achieved either by increasing the reservoir base aspect ratio *Γ* or by reducing the channel spacing *S*. However, when the reservoir base area was kept constant, increasing the aspect ratio resulted in a slight decrease in sperm concentration. This trend is likely due to discrepancies between the designed and fabricated geometries arising from the punching process. In elongated reservoir geometries, elastic deformation of the material may reduce the effective opening dimensions, resulting in a decrease in the actual reservoir base area. The fabricated geometry closely matched the designed geometry for a square reservoir (*Γ* = 1), whereas the deviation from the designed geometry increased as Γ increased and the reservoir became more elongated. This deviation results from geometry-dependent elastic deformation of the material during the punching process rather than device-to-device fabrication variability. Preliminary experiments using tracer microparticles support this interpretation. For example, a device with *Γ* = 5.43 exhibited an approximately 13% reduction in the reservoir base area relative to the designed geometry, which corresponded to an approximately 13% increase in mean flow velocity within the microchannels. For a given volume difference, the reduction in effective reservoir area increases the hydrostatic head difference, resulting in a higher flow velocity. Consequently, the number of sperm capable of migrating upstream against the flow was reduced, thereby decreasing the recovered sperm concentration. These findings suggest that, from the perspective of maximising sperm recovery, reservoir geometries close to a square shape are preferable. Based on this design principle, the cross-shaped configuration represents an effective strategy because it increases the total number of channels while mitigating the adverse effects associated with elongated reservoir geometries.

Channel spacing * S* showed no significant effect on recovered sperm concentration under the study conditions. Therefore, reducing *S* is an effective strategy for increasing the channel numbers while maintaining the overall device footprint. However, an excessively small spacing relative to channel height may compromise structural integrity, indicating the need for an appropriate balance between channel density and mechanical stability. The volume difference between the reservoirs also plays a critical role. Smaller volume differences resulted in higher sperm concentrations, whereas an excessively small hydrostatic head may lead to flow instability, such as backflow, during device handling. Therefore, a trade-off between recovery efficiency and operational robustness must be considered. Channel cross-sectional dimensions are another important design factor. Increasing the cross-sectional area tends to enhance sperm recovery. However, maintaining the mean flow velocity within an optimal range requires an increase in channel length to preserve hydrodynamic resistance. This modification may result in a larger device footprint, particularly for cross-shaped configurations. Conversely, excessively small channel dimensions increase the risk of sperm clogging. Furthermore, maintaining the flow velocity within a range that induces rheotaxis is essential. Previous studies have shown that rheotaxis is most pronounced at shear rates of approximately $\:\dot{\gamma\:}=2\mathrm{\--}9\:{\mathrm{s}}^{-1}$(Kantsler et al. 2014), and the device should be designed to operate within this range. Increasing the number of channels beyond a certain count can significantly reduce the flow velocity, as shown in Fig. 3, thereby weakening the induction of rheotaxis. Taken together, these results indicate that optimal device design requires a comprehensive balance among key parameters, including the channel number *C*, channel width *W*, channel height *H*, channel length *L*, and volume difference Δ*V*, to simultaneously maximise sperm recovery and maintain effective rheotactic conditions.

A comparison of the experimental and numerical results indicates that sperm recovery in the proposed device is strongly influenced by thigmotaxis. Sperm that reach the collection reservoir via rheotaxis tend to accumulate near the bottom surface and corners, owing to boundary-following behaviour. (Nosrati et al. 2016). Consequently, a fraction of these sperm subsequently re-enters the microchannels. The simulations showed that for the basic configuration (*C* = 40), approximately 53% of the introduced sperm re-entered the channels. This re-entry behaviour explains why recovered sperm concentration did not increase proportionally with channel numbers. Representative microscopic observations of the cross-shaped MPSS device (*C* = 240) are provided in Supplementary Movie S1. In the representative observation, 155 sperm migrated from the microchannels into the collection reservoir, whereas 107 sperm subsequently re-entered the microchannels, corresponding to a re-entry ratio of approximately 69%. This experimentally observed behaviour is consistent with the numerical simulation and supports the proposed mechanism underlying the non-linear increase in recovered sperm concentration with increasing sorting time and channel numbers.

Because sperm exhibit thigmotaxis, the probability of encountering channel openings is higher in the cross-shaped configuration, where channels are connected on four sides, than in the basic configuration where channels are present on only one side. Consequently, the cross-shaped configuration exhibited increased sperm re-entry into the microchannels, reducing the rate of increase in recovered sperm concentration with channel number *C*, as observed in Fig. 6(d). Despite this effect, the cross-shaped configuration enabled a higher total number of channels to be accommodated within the same reservoir base area. By contrast, in the basic configuration, increasing the number of channels by elongating the reservoir (i.e., increasing *Γ*) was subject to geometric constraints. Consequently, as shown in Fig. 6(a), the cross-shaped configuration can achieve higher recovered sperm concentrations because more channels can be incorporated within the same reservoir base area. Numerical simulations also showed a slight decrease in recovered sperm concentration with increasing channel spacing. This trend may be attributed to preferential sperm accumulation near the corners owing to thigmotaxis. With larger channel spacings, some microchannels are positioned closer to the corners, making sperm accumulated in these regions more likely to re-enter the microchannels, resulting in a slight decrease in the recovered sperm concentration. By contrast, the experimental results did not exhibit a statistically significant dependence on the channel spacing. Similarly, the weak dependence on aspect ratio observed in the simulations may be attributed to preferential sperm accumulation near the reservoir corners owing to thigmotaxis. As the aspect ratio decreases (i.e., as the collection reservoir becomes closer to a square shape), more microchannels are positioned near the corners, increasing the probability of sperm accumulated in these regions to re-enter the microchannels. By contrast, the experimental trend is likely associated with discrepancies between the designed and fabricated geometries arising from the punching process. Overall, the numerical model developed in this study qualitatively reproduced the experimental trends and provided mechanistic insight into sperm accumulation and re-entry in the collection reservoir. Therefore, the present modelling framework can serve as a mechanistic and design-support tool for examining how changes in device geometry influence sperm transport and recovery. Future work will focus on investigating device geometries that suppress thigmotaxis-induced re-entry into microchannels, thereby improving sperm recovery efficiency. Further quantitative validation against experimental data will be required to improve the predictive capability of the model.

The proposed numerical model has several limitations. The ABM is a behaviour-based model rather than a physics-based model, and its predictive performance depends on experimentally determined input parameters describing sperm behaviour. In addition, sperm–sperm interactions were not considered in the simulations. Consequently, sperm may accumulate near corners and walls more readily than in actual experiments, potentially leading to an overestimation of sperm re-entry into the microchannels. Furthermore, the computational domain was limited to the collection reservoir, whereas sperm transport in the actual device is influenced by the entire microfluidic system, including the semen reservoir, microchannels, and collection reservoir. Future studies should incorporate sperm–sperm interactions and extend the computational domain to the complete microfluidic device to improve the predictive accuracy of the numerical model.

As summarized in Table S1, previously reported rheotaxis- and/or thigmotaxis-based microfluidic sperm selection devices have generally relied on syringe pumps to generate controlled flow fields. By contrast, the proposed MPSS operates solely by hydrostatic pressure, eliminating the need for external pumping equipment. The present device achieved a recovered sperm concentration of 2.3 × 10^6^ cells mL^− 1^, which is within the range reported for previous microfluidic sperm-sorting devices. However, in terms of recovery efficiency, the value obtained with the present device is relatively low (2.4 ± 0.3%). As shown in Table S1, this recovery efficiency is comparable to or higher than those reported for several rheotaxis- and/or thigmotaxis-based microfluidic devices, although higher recovery efficiencies have also been reported. Accordingly, the relatively low overall recovery efficiency remains an important limitation of the present device. As demonstrated by the experimental and numerical analyses, sperm re-entry from the collection reservoir into the microchannels is one factor limiting net sperm recovery. Further optimisation of the device geometry to suppress sperm re-entry will therefore be necessary to improve recovery efficiency while maintaining a sperm concentration appropriate for the intended application.

One of the potential applications of the present device is sperm preparation for bovine IVF. In conventional bovine IVF, the final sperm concentration during insemination is typically adjusted to approximately 1–2 × 10^6^ sperm/mL, and oocytes are commonly inseminated in droplets of approximately 50–100 µL (Ferré et al. 2020). Maximising the sperm concentration is not necessarily desirable because increasing the sperm concentration beyond an appropriate range can increase the incidence of polyspermic fertilisation, which may adversely affect subsequent embryo development (Parrish et al. 2014). The sperm concentration obtained with the present device (approximately 2.3 × 10^6^ sperm/mL) is close to the concentration range commonly used for bovine IVF and can be readily adjusted to the desired insemination concentration. Therefore, the sperm concentration achieved with the present device is quantitatively compatible with the concentration and volume scale required for conventional bovine IVF. These considerations indicate that achieving the highest possible recovered sperm concentration is not necessarily the most relevant performance criterion for this application; rather, obtaining selected sperm at an appropriate concentration for fertilisation is most important.

A limitation of the present study is the relatively small number of independent experiments (*N* = 4). To improve the transparency of the statistical analyses, detailed results of the normality tests, homogeneity of variance tests, statistical analyses, and effect sizes are provided in Table S2 and S3. Although the assumptions of normality and homogeneity of variance were satisfied and statistically significant differences were observed, future studies with a larger number of independent experiments are warranted to further validate the robustness and reproducibility of the present findings. A direct comparison between the MPSS and conventional DGC will also be important to establish the relative performance of the proposed method. Future studies should systematically compare these methods in terms of recovery performance, sperm concentration, motility parameters, and sperm quality, including DNA fragmentation, as well as further biological outcomes such as fertilisation potential and embryo development. Although the present study employed a sorting time of 30 min to enable a direct comparison with the conventional sperm preparation procedure, reducing the sorting time is an important consideration for practical application. Accordingly, future studies will focus on improving the design and operating conditions of the MPSS to shorten the sorting time while maintaining sperm recovery performance, thereby enabling more rapid sperm preparation.

Conventional sperm selection methods, such as DGC, require dedicated equipment, including centrifuges, resulting in non-negligible equipment and operational costs. By contrast, the proposed device operates via a passive mechanism driven by a hydrostatic pressure difference and does not require external actuation systems such as syringe pumps or centrifuges. This pump-free and centrifugation-free operation simplifies the equipment required for sperm preparation and facilitates practical implementation. In this study, oxygen plasma treatment was employed to render the PDMS surfaces hydrophilic. Because PDMS is intrinsically hydrophobic, plasma treatment facilitates liquid filling and stable flow within the microchannels. However, the hydrophilic state gradually reverts to a hydrophobic state over time, typically within several hours. Although this limitation is not critical for short-term laboratory experiments, it may impose practical constraints on field applications in which plasma equipment is not readily available. From a practical implementation perspective, achieving long-term hydrophilicity is essential. Possible strategies include the use of commercially available surface-modification agents or durable surface coatings. Alternatively, replacing PDMS with inherently hydrophilic materials, such as thermoplastics or glass, may simplify fabrication and improve long-term stability. Future work should also focus on systematically evaluating material properties, flow stability, and potential effects on sperm to optimise material selection and surface treatment for real-world deployment.

## Conclusions

A hydrostatically driven microfluidic device was developed for non-invasive selection and recovery of highly motile sperm by leveraging rheotaxis and thigmotaxis. The device enabled stable microflow generation without external actuation and provides a simple and cost-effective platform for sperm sorting. Systematic experimental and numerical analyses demonstrated that sperm recovery was strongly influenced by geometric and hydrodynamic parameters. Increasing the number of microchannels improved the recovered sperm concentration, whereas an excessive number of channels reduced the flow velocity and weakened the rheotactic guidance. A cross-shaped configuration enabled a higher total number of channels within the same reservoir base area and enhanced overall sperm recovery, although thigmotaxis-induced sperm re-entry into the microchannels resulted in non-linear scaling behaviour. The numerical model successfully reproduced the qualitative experimental trends and provided mechanistic insights into sperm accumulation near surfaces and subsequent re-entry into the microchannels.

These findings indicate that simply increasing the number of microchannels does not necessarily improve sperm-sorting performance because excessive channel density promotes sperm re-entry into the microchannels. Therefore, the design of hydrostatically driven sperm-sorting devices should achieve an appropriate balance between the number and arrangement of microchannels and the resulting flow conditions to maximise recovery performance while minimizing sperm re-entry. The proposed device provides a pump-free approach to sperm selection that avoids centrifugation-associated processing. Future work will focus on suppressing thigmotaxis-induced sperm re-entry to improve recovery performance and evaluating the fertilisation potential and subsequent embryo development of sperm selected using the MPSS.

## Supplementary Information

Below is the link to the electronic supplementary material.

Supplementary Material 1

Supplementary Material 2

Supplementary Material 3

Supplementary Material 4

Supplementary Material 5

## Data Availability

All data supporting the findings of this study are available upon reasonable request from the corresponding author.
