# Supplementary material for: Hydrostatically driven microfluidic sorting of highly motile sperm using multiple parallel microchannels via rheotaxis and thigmotaxis
Source: Biomed Microdevices. 2026 Oct 29;28(4):74. doi: 10.1007/s10544-026-00858-2 (PMC13723625; doi:10.1007/s10544-026-00858-2)
Supplement: Supplementary file 1 [file 10544_2026_858_MOESM2_ESM.docx]

**Supplementary Table S3. Statistical analysis of the effects of geometric parameters on the recovered sperm concentration shown in Figure 5.**

| **Experiment** | **Groups** | **Group means ± SD** | **Shapiro–Wilk test p-value** | **Levene’s test p-value** | **ANOVA p-value** | **η²** |
| --- | --- | --- | --- | --- | --- | --- |
| Initial volume difference  (Δ*V* = 20 μL) | *C* = 40 | 0.315 ± 0.073 | 0.734 | 0.081 | *p* < 0.000 | 0.883 |
|  | *C* = 80 | 0.723 ± 0.174 | 0.891 |  |  |  |
|  | *C* = 120 | 1.025 ± 0.095 | 0.408 |  |  |  |
| Initial volume difference  (Δ*V* = 40 μL) | *C* = 40 | 0.205 ± 0.013 | 0.572 | 0.052 | 0.003 | 0.721 |
|  | *C* = 80 | 0.392 ± 0.070 | 0.999 |  |  |  |
|  | *C* = 120 | 0.717 ± 0.253 | 0.522 |  |  |  |
| Channel spacing | *S* = 17 μm | 0.764 ± 0.199 | 0.23 | 0.942 | 0.936 | 0.015 |
|  | *S* = 50 μm | 0.723 ± 0.174 | 0.891 |  |  |  |
|  | *S* = 100 μm | 0.761 ± 0.166 | 0.495 |  |  |  |
| Reservoir aspect ratio | *Γ* = 1 | 0.374 ± 0.032 | 0.105 | 0.505 | 0.335 | 0.216 |
|  | *Γ* = 2.34 | 0.340 ± 0.046 | 0.574 |  |  |  |
|  | *Γ* = 5.43 | 0.315 ± 0.073 | 0.734 |  |  |  |
| Channel number comparison | *C* = 40 | 0.315 ± 0.073 | 0.734 | 0.167 | 0.000 | 0.885 |
|  | *C* = 160 | 1.282 ± 0.229 | 0.252 |  |  |  |
|  | *C* = 240 | 1.989 ± 0.501 | 0.799 |  |  |  |
|  | *C* = 320 | 2.317 ± 0.315 | 0.537 |  |  |  |

Note. All group comparisons were performed using one-way analysis of variance (ANOVA). Effect sizes are reported as η².
