# Supplementary material for: Hydrostatically driven microfluidic sorting of highly motile sperm using multiple parallel microchannels via rheotaxis and thigmotaxis
Source: Biomed Microdevices. 2026 Oct 29;28(4):74. doi: 10.1007/s10544-026-00858-2 (PMC13723625; doi:10.1007/s10544-026-00858-2)
Supplement: Supplementary file 2 [file 10544_2026_858_MOESM3_ESM.docx]

**Table S1. Comparison of representative microfluidic sperm sorting devices employing rheotaxis and/or thigmotaxis.** Values not explicitly reported in the original articles were calculated from the published experimental data.

| **Study (Year)** | **Sorting mechanism** | **Driving method** | **Processing time (min)** | **Recovery volume (µL)** | **Recovered sperm concentration (×10⁶ cells mL⁻¹)** | **Total recovered sperm (×10⁴ cells)** | **Recovery efficiency (%)** |
| --- | --- | --- | --- | --- | --- | --- | --- |
| Sarbandi et al. (2021) | Rheotaxis | Syringe pump | 20 | 2.5 | 3.68ᵃ | 0.92ᵃ | 11.6ᵇ |
| Sharma et al. (2022) | Rheotaxis | Syringe pump | 60 | 70–80 | 0.14–0.99ᵃ | 0.98–7.9ᵃ | 1.38 ± 0.97 |
| Zeaei et al. (2023) | Rheotaxis and thigmotaxis | Syringe pump | 20 | 27 | 0.60ᵃ | 1.6 | 1.33–1.89ᵃ |
| Sharafatdoust et al. (2024) | Rheotaxis and thigmotaxis | Syringe pump | 20 | 4.8–6.2ᵃ | 4.10 | 2.0–2.6ᵃ | 0.49–0.64ᵃ |
| Puri et al. (2026) | Rheotaxis and thigmotaxis | Syringe pump | 20 | 100 | 2.60 | 26ᵃ | 8.1 ± 1.0 |
| **Present study** | **Rheotaxis and thigmotaxis** | **Hydrostatic pressure** | **30** | **100** | **2.30** | **23** | **2.4 ± 0.3** |

ᵃ Calculated from values reported in the original article.

ᵇ Sum of the reported net isolated percentages in Zones 1–4; actual post-retrieval recovery efficiency was not reported.
