# Supplementary material for: Hydrostatically driven microfluidic sorting of highly motile sperm using multiple parallel microchannels via rheotaxis and thigmotaxis
Source: Biomed Microdevices. 2026 Oct 29;28(4):74. doi: 10.1007/s10544-026-00858-2 (PMC13723625; doi:10.1007/s10544-026-00858-2)
Supplement: Supplementary file 3 [file 10544_2026_858_MOESM4_ESM.docx]

**Supplementary Table S2. Statistical analysis of comparisons between the semen reservoir and the collection reservoir for the sperm motility parameters presented in Figure 4.**

| **Parameter** | ***C*** | **Semen reservoir (mean ± SD)** | **Collection reservoir (mean ± SD)** | **Shapiro–Wilk test p-value** | **Levene’s test p-value** | **Student’s t-test p-value** | **Cohen’s d** |
| --- | --- | --- | --- | --- | --- | --- | --- |
| VCL [µm s^-1^] | 40 | 89.195 ± 7.915 | 141.008 ± 22.475 | 0.326 | 0.151 | 0.042 | 1.711 |
|  | 80 | 85.170 ± 5.844 | 130.580 ± 15.349 | 0.992 | 0.957 | 0.038 | 1.778 |
|  | 120 | 84.820 ± 6.285 | 119.284 ± 9.841 | 0.393 | 0.631 | 0.012 | 2.768 |
| STR [-] | 40 | 0.528 ± 0.026 | 0.734 ± 0.044 | 0.472 | 0.393 | 0.002 | 4.849 |
|  | 80 | 0.515 ± 0.037 | 0.704 ± 0.069 | 0.618 | 0.317 | 0.032 | 1.894 |
|  | 120 | 0.510 ± 0.039 | 0.652 ± 0.073 | 0.728 | 0.421 | 0.042 | 1.706 |
| BCF [Hz] | 40 | 7.345 ± 0.228 | 12.297 ± 1.764 | 0.236 | 0.072 | 0.008 | 3.136 |
|  | 80 | 7.547 ± 0.174 | 12.785 ± 1.489 | 0.751 | 0.080 | 0.006 | 3.603 |
|  | 120 | 7.430 ± 0.515 | 10.881 ± 0.985 | 0.818 | 0.275 | 0.004 | 4.080 |
| ALH [µm] | 40 | 2.467 ± 0.087 | 2.424 ± 0.287 | 0.970 | 0.171 | 0.763 | -0.165 |
|  | 80 | 2.328 ± 0.387 | 2.559 ± 0.376 | 0.856 | 0.882 | 0.519 | 0.365 |
|  | 120 | 2.310 ± 0.224 | 2.578 ± 0.232 | 0.534 | 0.978 | 0.214 | 0.786 |
